# Bridging the Gap: The Critical Role of Regulatory Affairs and Clinical Affairs in the Total Product Life Cycle of Pathology Imaging Devices and Software

**DOI:** 10.3389/fmed.2021.765385

**Published:** 2021-11-17

**Authors:** Staci J. Kearney, Amanda Lowe, Jochen K. Lennerz, Anil Parwani, Marilyn M. Bui, Katy Wack, Gina Giannini, Esther Abels

**Affiliations:** ^1^Elevation Strategic Development, Morrison, CO, United States; ^2^Visiopharm A/S, Horsholm, Denmark; ^3^Department of Pathology, Massachusetts General Hospital/Harvard Medical School, Center for Integrated Diagnostics, Boston, MA, United States; ^4^Wexner Medical Center, The Ohio State University, Pathology and Biomedical Informatics, Columbus, OH, United States; ^5^Department of Pathology, Moffitt Cancer Center and Research Institute, Tampa, FL, United States; ^6^PathAI, Boston, MA, United States; ^7^Roche Tissue Diagnostics, Tucson, AZ, United States

**Keywords:** regulatory strategy, clinical affairs, total product life cycle, *in vitro* diagnostics development, digital pathology, business strategy, artificial intelligence, software development

## Abstract

Manufacturers of pathology imaging devices and associated software engage regulatory affairs and clinical affairs (RACA) throughout the Total Product Life Cycle (TPLC) of regulated products. A number of manufacturers, pathologists, and end users are not familiar with how RACA involvement benefits each stage of the TPLC. RACA professionals are important contributors to product development and deployment strategies because these professionals maintain an understanding of the scientific, technical, and clinical aspects of biomedical product regulation, as well as the relevant knowledge of regulatory requirements, policies, and market trends for both local and global regulations and standards. Defining a regulatory and clinical strategy at the beginning of product design enables early evaluation of risks and provides assurance that the collected evidence supports the product's clinical claims (e.g., in a marketing application), its safe and effective use, and potential reimbursement strategies. It is recommended to involve RACA early and throughout the TPLC to assist with navigating changes in the regulatory environment and dynamic diagnostic market. Here we outline how various stakeholders can utilize RACA to navigate the nuanced landscape behind the development and use of clinical diagnostic products. Collectively, this work emphasizes the critical importance of RACA as an integral part of product development and, thereby, sustained innovation.

## Introduction

Pathology is the cornerstone of patient care, providing diagnostic, prognostic, and therapy-predictive information to a health care team. In the era of precision medicine and digital health, digital pathology tools and applications, including artificial intelligence (AI)-based applications, are enabling pathologists to deliver high-quality care to patients. However, more innovation is needed. The delivery of high-quality care to patients continues to become more and more complex in the rapidly evolving age of digital health, personalized medicine, and value-based care. Digital pathology, both hardware and software, is no exception. Streamlining the regulatory process to get innovative digital tools into the hands of practicing pathologists is in the best interest of patients.

Manufacturers of pathology imaging devices and associated software should engage regulatory affairs and clinical affairs (RACA) professionals throughout the total product life cycle (TPLC) of these innovative medical devices. A significant number of manufacturers and end users (e.g., practicing pathologists), especially in the digital health space, are unfamiliar with the TPLC for regulated products, the role of RACA in the product development process, and the rigor of bringing a medical device to market. These manufacturers, as well as pathologists as the end users, can utilize RACA professionals to help navigate the nuances behind development and use of a regulated product.

Here we try to increase awareness of the importance of the role of RACA in delivering these products to practicing pathologists, administrators, and developers by the following:

- Demystify RACA by describing how the regulatory landscape shapes the delivery of clinical products
- Bridge the gap between the mindsets of the developer and the end user on the implementation of regulatory requirements and product features
- Establish a mutual vocabulary to facilitate understanding of the application of regulatory requirements

## The Dynamic Regulatory Environment for Digital Pathology

Regulatory trends and expectations for the approval process and post-market responsibilities shift and evolve, sometimes rapidly, particularly for advanced technologies like digital pathology. Digital pathology products, both hardware and software, are regulated as *in vitro* diagnostics (IVDs). From 2019 to 2020 alone, the US Food and Drug Administration (FDA) published more than 40 draft and final guidances that impact software, digital pathology, and IVD product development or approval [this does not include guidance specific to Coronavirus Disease 2019 (COVID-19)] (1). Also in 2020, members of Congress introduced the Verifying Accurate, Leading-edge IVCT Development (VALID) Act (2), which, if passed, will fundamentally change the regulation of diagnostic tests in the US. Similarly, the European Union (EU) introduced a transformative set of regulations for IVDs in 2017, with the entry into force of the *in vitro* diagnostic device regulation (IVDR) (3). Full compliance with the IVDR will be required in May 2022.

Transformational healthcare initiatives by regulatory bodies must also be considered. FDA's Digital Health Initiative outlines efforts to reimagine FDA's approach to ensuring timely access to high-quality, safe, and effective digital health products (4). It also encourages innovation and the facilitation of new approaches that support health care delivery and sharing of information. FDA is also a key participant in the Precision Medicine Initiative launched in January 2015 (5). Precision medicine, sometimes known as “personalized medicine,” is an innovative approach to tailoring disease prevention and treatment that considers differences in people's genes, environments, and lifestyles. The goal of precision medicine is to target the right treatments to the right patients at the right time.

The success of precision medicine depends on having accurate, reproducible, and clinically useful diagnostic tests, including companion diagnostic (CDx) tests to identify patients who can benefit from targeted therapies. The diagnosis of breast cancer is a very good example. Four primary biomarkers are analyzed during the routine pathological work-up for breast cancer: estrogen receptor (ER), progesterone receptor (PR), human epidermal growth factor receptor 2 (HER2), and the proliferation-associated nuclear protein Ki67. Assessments of these biomarkers within collected tumor tissue (e.g., biopsy or surgical resection) are combined into surrogate subtype classifications, guiding conclusions about the tumor's biological characteristics and expected response to therapy. A comprehensive study conducted in 2016 by the Department of Clinical Pathology at Karolinska University Hospital in Stockholm, Sweden, demonstrated that image analysis performed on whole slide images (WSIs) of tissue on glass slides was superior to manual assessment and provided more prognostic information than the manual scores (6). The results of this study have been repeated numerous times, demonstrating that digital pathology products will be important for expanding the potential of diagnostic tests.

The complex and dynamic nature of medical device development requires engagement of multiple cross-functional disciplines. RACA professionals are an important contributor to product development and deployment strategies because they must maintain an understanding of the scientific, technical, and clinical aspects of a biomedical product, as well as deep knowledge of regulatory requirements, policies, and trends for both local and global regulations and standards. For this reason, it is recommended that RACA be involved very early in the TPLC to assist with navigating changes in the regulatory environment and the market. RACA can also optimize business processes for the TPLC through this knowledge sharing.

## RACA Defined

RACA professionals work to design and promote a regulatory strategy that is aligned to the current regulatory landscape for a given clinical product or products. The strategy focuses on the efficacy and safety of the product(s), without sacrificing quality, and while ensuring an efficient time to market.

Regulatory affairs (RA) is often recognized for its role in communication with health authorities and overseeing regulatory submissions; however, RA's core competency is developing strategies that comply with both global and local regulations and standards, which are often moving targets. RA is also responsible for gathering and effectively applying regulatory intelligence throughout the TPLC, including pre-market and post-market strategies (Table 1).

**Table 1:** Terminology defined: market environments.

**Term**	**Definition**
Regulatory intelligence	Information collected on the current regulatory environment and trends for an identified market.
Pre-market	A medical device is considered pre-market before it is offered commercially, which is typically during development. Review and clearance or approval of a marketing application is often required by a health authority for use in a clinical setting.
Post-market	A medical device is considered post-market when a manufacturer offers the product commercially. A product is marketed illegally if it is provided commercially without meeting applicable regulatory requirements.

*A medical device is a regulated product regardless of whether it is used only for research or in a clinical setting*.

Clinical affairs (CA) includes clinical science, strategy, and clinical operations; it is responsible for the generation and dissemination of sound scientific and clinical evidence, such as clinical study protocols, reports, and publications. Often, CA is key in defining clinical strategies that support a company's development objectives, while ensuring that products are designed according to a robust clinical evidence strategy. The generation of clinical evidence not only supports the product's introduction to the market, but also provides the foundation for establishing reimbursement strategies, which can drive the economic value of the product to the company. Carrying out successful clinical studies for development requires cooperation between CA and multiple functional groups, such as data managers, biostatisticians, business development, information technology, RA, research coordinators, product management/engineering, and many other functions.

While RA and CA have distinct responsibilities, these roles often overlap in the development of a product and in influencing and shaping the regulatory landscape. Importantly, RACA can bring together the developer and the user, including the patient, through external-facing roles that foster relationships that can be beneficial to the perception and adoption of the product. RACA can also assist with prioritizing markets based on the clinical and regulatory landscape, as well as ensure the correct intended use is identified and the supporting clinical evidence generated to align to the market environment. Overall, RACA provides a critical role in the product TPLC by applying clinical and regulatory strategies that can reduce business risk and product risk at all phases of development and commercialization.

RACA professionals' influence is often achieved through collaborations with regulatory bodies to drive policy and application of regulations, as well as through work to design and implement innovative approaches. To achieve this, RACA professionals are often contributors to technical committees, consortia, and trade organizations that work to accelerate standard development or to improve standards or guidelines to be more compatible with current technologies. For example, in 2016, the Digital Pathology Association's (DPA's) Regulatory and Standards Task Force played a major role in getting the device classification for WSI systems reclassified from an automatic Class III medical device that requires submission of a pre-market approval application (PMA) to a Class II device *via* a *de novo* request (7). The DPA and FDA closely collaborated on introducing consistency in terminology and developing general principles for test protocols that were acceptable to FDA. This close collaboration between regulators, healthcare workers, medical specialists, and industry represented a major shift in FDA's approach to WSI systems. Continued communication between FDA and digital and computational pathology-enabled organizations like the DPA, Association for Pathology Informatics (API), and the Pathology Innovation Collaborative Community (PIcc, formerly known as Alliance for Digital Pathology) is taking place to address AI-related products in pathology.

## Recommendations on the Use of RACA in the TPLC

**Figure 1:**
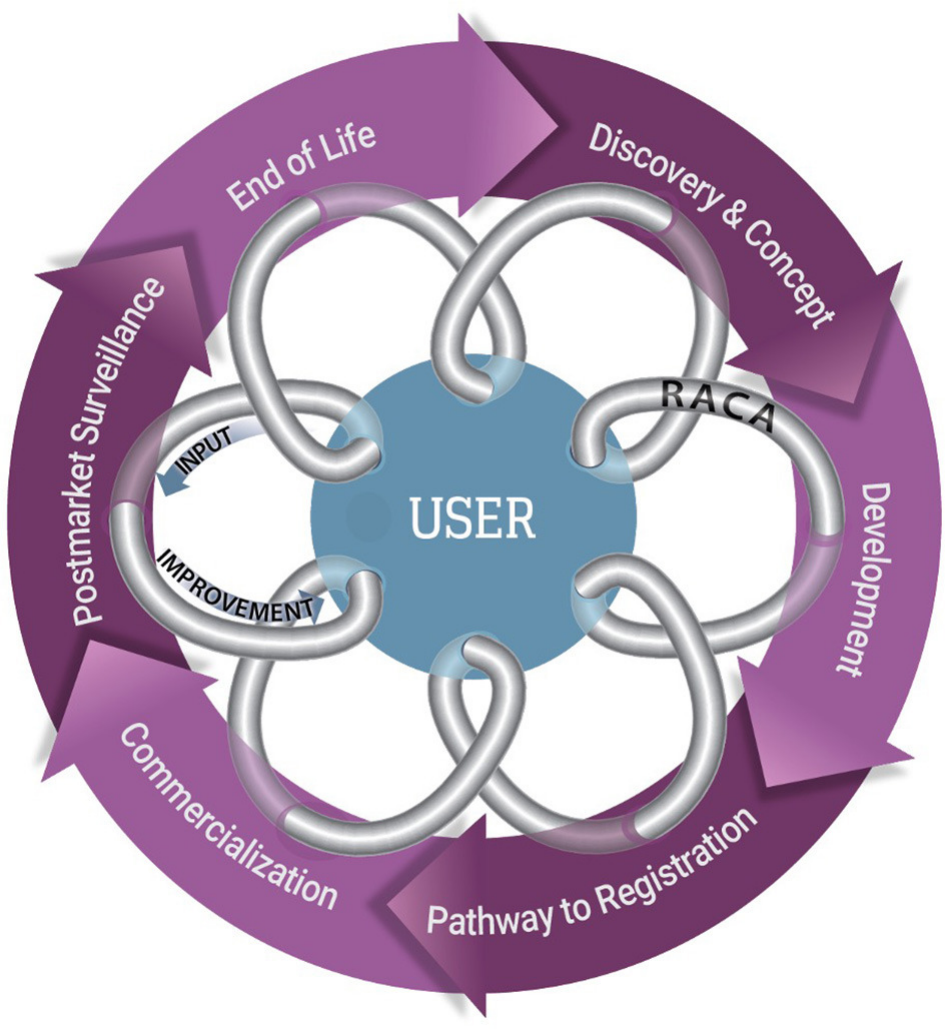
The feedback loop for the TPLC for a commercialized clinical product. The TPLC for a commercialized clinical product, in general includes 6 primary steps that are often iterative in nature (purple circle). Manufacturers benefit from seeking input from users, such as pathologists and other healthcare providers, to continually introduce improvements and utility (arrows) into the product design. RACA professionals can be a conduit between users and industry (gray coil) to assist with delivering user input and communicating the implications of the changes.

Medical device development is often an iterative process; in general, it includes device discovery and concept, development, pathway to registration, commercialization, post-market surveillance, and end of life (Figure 1). RACA uses market and regulatory intelligence to work with product management and development teams to define clinical utilization, which provides clarity on the user requirements and formulation of the intended use, indications of use, and claim definitions. These descriptions then drive the device description, device classification, if applicable, and regulatory pathway.

Similarly, RACA engages clinical domain experts, who are users of the products, which often includes a collaboration with a field sales team. RACA can acquire input from users on product utility and function independently or with the help of product management. This is input is typically gathered through focus groups, surveys, and one-on-one interviews and is critical for ensuring a design that provides a safe, effective, commercially viable, and high-quality product. Additionally, this input, together with the state of the art of the product and comparison to standard of care, determines the benefit risk ratio used in submissions to regulatory authorities. While RACA professionals often seek out these clinical domain experts to receive their input, end users can engage the RACA professionals on the products they use typically through a customer support or sales channel of the product manufacturer.

### Discovery and Concept

RACA can represent an aligning element in the product design and concept generation process. The design of a product should include screening the possible regulatory opportunities and risks based on a company's vision of a product and its geographical regions of deployment. This screening includes working within the product management and development team to identify opportunities, competition, development trends, and avenues for deployment. The screening process also provides an opportunity to develop insights for shaping the architectural design and intended use to suit most markets.

To initiate the screening process, manufacturers must first generate a technical device description and architecture and then build requirements around the intended use according to the description. This intended use, technical device description, and architecture will drive the regulatory pathway and requirements needed to develop the product, which can vary from region to region. Using IVDs within the US and the EU as an example, device classifications, approval pathways, required supporting evidence, and post-marketing responsibilities differ. In the US, a medical device must comply with Code of Federal Regulations Title 21 Part 820 (21 CFR 820), under which medical devices and IVDs are not defined as separate (i.e., both are regulated under 21 CFR 820). However, in the EU, medical devices and IVDs are regulated under separate directives, the Medical Device Directive (MDD) 93/42/EEC and the *in vitro* diagnostic directive (IVDD) 98/79/EC, respectively. As of 2017, the EU entered into force the IVDR (2017/746/EC), which represents a large change from the previous directive (3). Under the IVDR, there are 4 IVD classifications (Classes A, B, C, and D), while medical devices in the US only have 3 classifications (Class I, Class II, and Class III). Using RACA professionals to understand how the requirements for each regulation are different and similar may allow a company to introduce efficiencies in development that meet both, while ensuring region-specific requirements are also met.

The interaction between RACA professionals and the commercialization team, including product management, sales, and marketing, is also essential at this early phase of product development to ensure that messaging and target markets are properly reached. This includes a profiling of the product to assess the overall product and business risks, which includes but is not limited to segmentation of the market, possible reach of market, the regulatory paths per target market, opportunities to obtain reimbursement including level of evidence required per market and related efforts and timelines. This profiling is an essential input for pricing and business models to enable the business to decide whether the product will be developed, brought to market (a so called “GO” or “NO GO” decision), and how it will be brought to the market.

### Development

Device development is often an iterative process that includes refining or even changing a device design as the concept becomes a functioning prototype and then a finished device. Device design documentation is used to record the development process, usually by multiple stakeholders. This documentation relates to a series of development activities, including product requirements, market research, and customer input, as well as verification, validation, and clinical performance studies. Regulatory/health authorities often require products to meet certain specifications before the medical device can be deployed for its intended use.

While the specifics of development requirements can differ from region to region, the general principles of design control are a well-established and recognized standard. ISO 13485 is an internationally recognized standard that specifies requirements for a quality management system (QMS) to support the design and manufacturing of medical devices, including design controls (8). While the US has its own regulations that define requirements for design controls (21 CFR 820.30), they are highly similar to the internationally recognized principles (e.g., ISO 13485). However, it has been increasingly recognized that the design principles for software applications, such as for AI and machine learning (ML) applications for digital pathology, require a unique development approach that is more tailored to this type of technology. While the foundational design tenets are applied, software documentation, testing, traceability, and configuration management can be conducted and even scrutinized differently than non-software medical devices. International standards for software development exist [e.g., IEC 62304: Medical Device Software–Software Life Cycle Processes (9) and IEC 82304-1: Health Software (10)], but the landscape continues to evolve as the introduction of AI/ML-based software as a medical device (SaMD) and its applications rapidly expand.

In addition to a regulatory strategy, the requirements for clinical evidence should be identified early so worldwide clinical studies can be planned. Defining a clinical strategy at the beginning of product design allows for early evaluation of clinical risks and provides assurances that the clinical evidence could support validation of the product clinical claims in a marketing application. For manufacturers of digital pathology products, there are endless strategy and product design parameter combinations to consider: tissue, disease, biomarker, WSI digital scanner, viewing system, and display, as well as the operational environment. To take each factor into consideration, the appropriate design of clinical studies requires a well-established network of collaboration within the biomedical field, such as with key opinion leaders (KOLs), who are often the end user of the product; contract research organizations (CROs); and development partners. Experienced RACA professionals can facilitate bringing these resources to the development process.

### Pathway to Registration

National and international regulations provide guidelines to manufacturers regarding the regulatory pathway and registration procedures for medical devices, which must be followed prior to the sale and marketing of the device for diagnostic use in the intended clinical market. Within each global region, the regulatory pathway to device registration is defined by the classification of the device. For example, in the US, Class II medical devices are either cleared through a 510 (k) pre-market notification submission or a *de novo* request is granted (Table 2). Class III medical devices are approved through submission and review of a PMA application. All of these pathways have widely different requirements for evidence, including clinical evidence and documentation to support review of the submission. As previously noted, passing of the VALID Act will change the classification and approval process in the US, which will require significant preparation by manufacturers. Similarly, in the EU, an IVD's classification determines the appropriate conformity assessment procedure to follow and whether a notified body (NB) will be involved in the registration and certification process. The change in classifications in the IVDR dramatically increases the conformity requirements, and NBs will typically be more involved than in the past. For example, the burden of the TPLC for Class C and D devices is much higher than for Class A and B because these devices require at least annual updating of the performance evaluation report. As noted above, the intended use drives the device classification, and RACA input can be critical to ensuring the intended use language does not unintentionally place the device in a higher class than is needed for its clinical use. If this occurs, it could have a large impact on the business case for the product and cause the burden of development to exceed the market share or opportunity.

**Table 2:** Terminology defined: US regulatory pathways.

**Term**	**Definition**
Cleared	FDA *clears* a medical device to be marketed after a manufacturer submits a 510 (k) marketing application and demonstrates substantial equivalency to a predicate device, as well as follows general controls, such as good manufacturing practices and special controls. This is a Class II medical device submission pathway.
*De novo* request granted	Manufacturers submit a *de novo* request (i.e., the marketing application) for Class II medical devices for which there is no predicate device. Upon review, FDA grants the request for the medical device to be a Class II device, and the medical device is considered cleared for marketing. This pathway is typically more rigorous than the 510 (k) pathway, but less rigorous than that for a Class III device.
Approved	Class III medical devices go through a rigorous and substantial review when manufactures submit a PMA application for FDA review. When a device is found to be safe and effective, FDA *approves* it for marketing.

*Specific terminology defines which FDA submission pathway a medical device has gone through. For manufacturers, each term has implications for device development*.

RACA professionals are becoming key members of the development team who assist with managing risk due to changing requirements. To de-risk the regulatory review process and understand expectations for evidence and documentation prior to submission of a marketing application, developers can seek feedback from FDA through the Pre-Submission Program (11). Within this program, a Q-Submission can be provided to FDA that includes a formal written request from a developer for feedback from FDA on development plans that is provided in the form of a formal written response or, if the submitter chooses, formal written feedback followed by a meeting in which any additional feedback or clarifications are documented in meeting minutes. Through these communications, a developer has the opportunity to obtain FDA feedback prior to submission of a marketing application. These communications are entirely voluntary on the part of the developer, but early interaction with FDA on planned non-clinical and clinical studies and careful consideration of FDA's feedback may improve the quality of subsequent submissions, shorten total review times, and facilitate the development process for new devices.

RACA typically takes the lead in organizing communications with regulatory bodies, including the strategy for how and when to gather information on a particular stage of development. A formalized program like FDA's Pre-Submission Program is less pronounced in other regions of the world but, in the EU, manufacturers can use engagement with NBs to understand certification requirements. NBs are required to be designated for IVDR to perform conformity assessments under this regulation. To date, there are only 6 designated NBs for IVDR (12), which is causing some concern among manufacturers as the May 2022 compliance date rapidly approaches. RACA professionals are often responsible for contracting with NBs and, even more importantly, are responsible for making well-informed decisions about the concepts, content, and specific language used in an application based on the different policies and processes defined by each NB. This requires development of strong relationships with an NB and use of regulatory domain knowledge to incorporate technical and clinical information into an application for review. Therefore, RACA represents the gateway to the competent authorities.

An additional pathway to entry to the clinical market that is unique to IVDs is offering a clinical test as a laboratory developed test (LDT). An LDT is defined as a diagnostic that is designed, manufactured, and used within a single laboratory (13). In the US, LDTs can be offered as clinical tests under the Clinical Laboratory Improvement Amendments (CLIA) regulations without having gone through FDA clearance or approval (14). Numerous advanced diagnostics, such as next generation sequencing (NGS), flow cytometry, polymerase chain reaction (PCR), and histopathology image analysis applications, have been introduced into clinical use by this pathway. The COVID-19 public health emergency (PHE) highlighted the agility of this pathway. In March 2020, a Memorandum issued by the White House reversed FDA's position that all COVID-19 diagnostic tests must undergo an Emergency Use Authorization (EUA) review and approval by FDA, and instead allowed independent authorization of LDTs by states (15). This reversal was likely due to a need for expediency in the availability of and access to these tests in unprecedented circumstances and FDA's limited resources to handle the onslaught of submissions. However, controversy has long existed about the enforcement discretion FDA has applied to the regulation of LDTs. The introduction of the VALID Act will formally define the regulation of LDTs by FDA, which will require these types of developers to establish a compliant QMS and be subject to pre-market review, when applicable, for all tests offered for clinical use. Similarly, the new IVDR increases restrictions on the ability to offer LDTs in the EU.

### Commercialization

Approval/clearance of a regulatory submission or successful registration allow for commercial introduction into the clinical market but represents only the first step in the adoption of a product. A customer will adopt innovation more easily when the return on investment (ROI) is proven, and ROI can be influenced by payer reimbursements. This increases the need for early identification of reimbursement and other incentives for ROI for users and manufacturers. An example of using RACA experts to optimize the go-to-market strategy is leveraging their knowledge of health authority programs that can benefit product commercialization. FDA's Breakthrough Device Designation (BDD) program (16) is a voluntary program for certain devices that provides manufacturers prioritized review of a submission, shortening the time to market, and potentially as a benefit for reimbursement strategies based on the Medicare Coverage of Innovative Technology (MCIT) rule (17). While the process of obtaining reimbursement is outside the scope of this paper, it is valuable to use a RACA professional's unique knowledge and experience to provide insights about how to utilize regulatory intelligence and device information, such as the intended use, indications of use, claims, and clinical safety, within a commercialization strategy (Table 3).

**Table 3:** Terminology defined: regulated product information.

**Term**	**Definition**
Intended use/indications of use	Statements in the labeling that describe the purpose of the device, including the disease or condition for which the medical device can be used. Health authorities review and determine the appropriateness of these statements for inclusion in medical device labeling. The use of the medical device will be limited to the context of these statements.
Labeling	The information that identifies and describes a medical device. This can include the stickers or tags on the physical device, but also includes the instructions for use (IFU), which define how, when, and by whom a medical device can be used.
Claim	A statement about the safety, efficacy, or use of a medical device. Health authorities will only allow manufacturers to make claims that have been proven in marketing applications by evidence.

*The scope and context of use of a medical device are defined by a manufacture and captured in the device information. Health authorities review this information to determine its appropriateness based on the evidence provided, and the medical device use is then limited to the defined information*.

**Figure 2:**
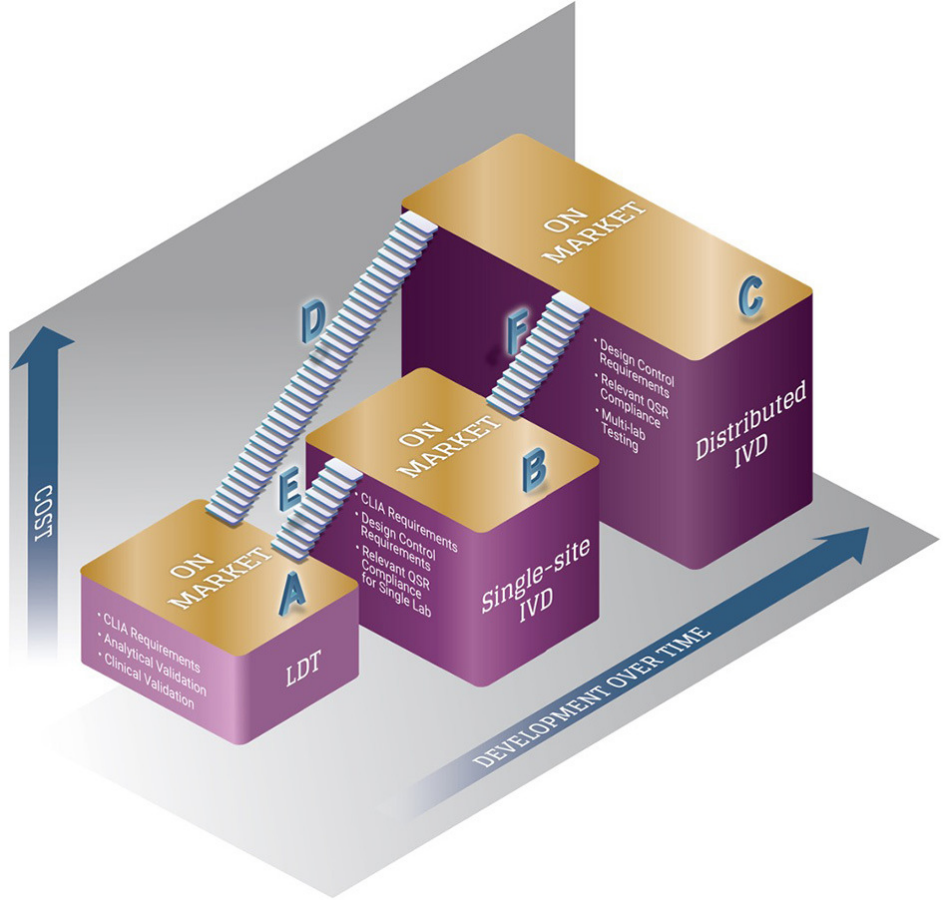
Multiple pathways exist for bringing a clinical diagnostic product to market in the US. The introduction of a clinical diagnostic product on the market can proceed through multiple pathways. As straight-to-market strategies (A, B, C), manufacturers who also have laboratories (e.g., CLIA-certified lab) can choose to introduce a diagnostic test as an LDT, which is currently regulated under CLIA (A) or as a single-site IVD, which undergoes FDA review (B). Manufacturers intending to distribute a diagnostic for broad use by clinical laboratories must complete multi-lab testing and usually represents the greatest compliance requirements, but also greatest access (C). A stepwise approach can also be utilized by manufacturers who have laboratories as a de-risking strategy of reaching multiple on-market milestones along the commercialization pathway (D, E, F). A clinical diagnostic test first offered as an LDT can then be further developed directly as a distributed IVD (D), or first developed with increasing requirements to undergo FDA review as a single-site IVD (E), and then further development for a distributed IVD (E + F). Some LDTs are only offered as a single-site IVD as a second milestone (E), which is typical for advanced diagnostics. Similarly, products first offered as a single-site IVD can then be further developed as a distributed version (F). Each approach has pros and cons and differing associated requirements, and the cost and development timeline for each approach should be considered in evaluating the pros and cons.

Labeling is also an important component of commercialization, which is actually a very broad term. Labeling can include instructions for use (IFU), packaging, all forms of advertisement, and any external communications or descriptions of the device. Labeling materials are typically generated by engineers and products teams, but RACA provides important input on the boundaries of labeling. For example, it is advisable for RACA to review all external communications that discuss the product, even those that might seem unrelated, such as an investor presentation. Word choice and descriptions must be considered carefully to prevent false advertising of the product that could unintentionally trigger consequences from regulatory authorities. RACA input can also be critical when determining how labeling can influence market positioning. For IVDs, products in the laboratory research phase can be labeled for research use only, making them exempt from most regulatory requirements, including pre-market requirements and/or applications (18). However, any reference to clinical claims or use could cause a product to become subject to regulatory oversight, which impacts sale and distribution of the product. RACA should be used to ensure this boundary is not inadvertently crossed. Gathering this input in the early phases of the TPLC can help optimize the approach for initial market entry of a product and beyond (Figure 2).

### Post-market Surveillance

A manufacturer's responsibility for monitoring safety in the use of a product does not end after the product's validation and approval/clearance/registration. The safe use of a medical device must be continuously monitored, and when indicated, the product must be recalled or redesigned to improve the safety profile. To increase the discovery of adverse events in the general population, FDA created a safety information and adverse event reporting service (AERS) called MedWatch. MedWatch is a voluntary reporting system for consumers, patients, and health professionals that allows for safety surveillance of medical devices, as well as other FDA-regulated products (19). The Manufacturer and User Facility Device Experience (MAUDE) database is an additional system for monitoring safety (20). MAUDE houses medical device reports submitted to FDA by mandatory reporters, including manufacturers, importers, and device user facilities. Reporting through these systems can sometimes lead to initiating a voluntary or involuntary recall of a product and/or additional testing, including a potential clinical study to determine if product changes are needed. It is recommended that RACA professionals take the lead in executing both activities.

Requiring commitments to conduct clinical studies in the post-market is an approach regulators can use to support continued approval of a product. For example, as a condition of approval, FDA can include a post-market commitment to conduct additional studies. These studies are often used to allow expedited access to a product, while continuing to gather information on its performance. Post-market studies also provide significant commercial value to support ROI and improvements made to a medical device. This approach has been applied with increasing frequency, particularly for expedited approval pathways, such as for the devices that receive a BDD from FDA. FDA grants a BDD for certain medical devices and device-led combination products that provide for more effective treatment or diagnosis of life-threatening or irreversibly debilitating diseases or conditions. It is available for devices and device-led combination products that are subject to PMA, 510 (k), or *de novo* request review. The BDD program is intended to help patients have more timely access to these medical devices by expediting their development, assessment, and review, while preserving the statutory standards for authorization (16). This path could be extremely beneficial when a device will be used as a CDx for a drug that has been granted accelerated approval. The first digital pathology BDD was issued in 2019 (21).

In certain regions, post-market studies can also be a requirement for any marketed clinical product. In the EU, manufacturers are required to establish post-market surveillance (PMS) plans to comply with the IVDR. The IVDR states that manufacturers must play an active role in gathering information in a way that allows for regular updates to technical documentation, including Post-market Performance Follow-up (PMPF) studies. Using RACA experts early in the TPLC will help to identify the least burdensome approach for the PMPF.

### End of Life

End of life for a product could be represented by retirement of an obsolete technology or, more commonly, change and improvement to an existing device that represents the next generation and eventual retirement of the previous version. For the latter, manufacturers have important considerations and requirements when applying changes and introducing an improvement, update, or even more substantial change to a medical device currently on the market. Specifically, a manufacturer must determine how to support previous versions of a medical device when a new version is introduced. These devices must be phased out appropriately to avoid interruptions in patient care for customers still utilizing the technology. This is also an important business consideration when the medical device is being used as a CDx. Once the drug is on the market, it could remain there forever and, if indicated, the CDx must continue to support the drug use. This could be challenging for devices that require frequent updates, such as SaMDs types of devices, and remaining compatible with rapid evolving technology could have business impacts.

Important consideration also must be given to how a new version of an existing product is introduced to the market, which will likely have requirements for additional regulatory submissions and, potentially, analytical and/or clinical studies. RACA professionals can assist with understanding when a product change has regulatory implications, which can differ depending on the technology and type of change. For example, FDA recognized in a recently published discussion paper that AI-based SaMD algorithms, which include those for digital pathology applications, should have appropriately tailored regulatory oversight to prevent unnecessary barriers to access to innovation (22). This discussion paper proposes potential approaches that could decrease certain requirements for submissions due to device changes in the post-market, such as approval of pre-determined change-control plans that include SaMD pre-specifications and an algorithm change protocol. FDA subsequently published an action plan for AI/ML-based SaMDs that outlines the actions FDA will take to develop this framework (23). Given the changing nature of the regulatory landscape for innovative technologies in the post-market and varying requirements for different technologies, RACA professionals can be vital to product teams in supporting product updates.

## Discussion

Digital pathology is relatively new to clinical diagnostic pathology, but the technology has been used extensively for research over the past 20 years (24). The safety of patients and the quality of the pathology results are critical to the practice of pathology, which is highly controlled by various regulatory bodies. However, it should be noted that pathology as a medical practice is not under the authority of a regulatory body. Based on the intended use of digital pathology devices to date, whole slide scanners, viewers, image management systems (IMSs), and algorithms are classified as one or more types of medical devices (Table 4). Numerous digital pathology devices have been cleared in the last few decades, including more than 20 devices over the last 10 years (Table 5).

**Table 4:** Product codes and descriptions for digital pathology devices.

**Product code**	**Description**	**Regulatory number**
OEO	Automated digital image manual interpretation microscope	21 CFR 864.1860
NQN	Microscope, automated, image analysis, immunohistochemistry, operator intervention, nuclear intensity, and percent positivity	21 CFR 864.1860
NOT	Microscope, automated, image analysis, and operator intervention	21 CFR 864.1860
PSY	Whole slide imaging system	21 CFR 864.3700
PZZ	Digital pathology display	21 CFR 864.3700
QKQ	Digital pathology image viewing and management software	21 CFR 864.3700

**Table 5:** FDA cleared digital pathology devices within the last 10 years.

510 (k) number^*^	**Product**	Product code^**^	**Approval date (mm/dd/yyyy)**
K111543	VIRTUOSO (TM) System for IHC HER2 (4B5)	OEO; NOT	10/12/2011
K111755	VIRTUOSO System for IHC KI-67 (30-9)	OEO; NQN; NOT	02/22/2012
K111869	VIRTUOSO System for IHC PR (IE2)	OEO; NQN	03/05/2012
K111872	VIRTUOSO System for IHC P53 (DO-7)	OEO; NQN; NOT	04/19/2012
K111914	Virtual Slide System Olympus VS800 System	OEO	08/21/2012
K121033	VIRTUOSO System for IHC KI-67 (30-9)	OEO; NQN; NOT	09/6/2013
K121350	VIRTUOSO System for IHC (DO-7)	OEO; NQN; NOT	06/01/2012
K121516	VIRTUOSO System for IHC HER2 (4B5)	OEO; NQN; NOT	09/26/2013
K122143	VIRTUOSO System for IHC PR (1E2) Benchmark Ultra Stainer	OEO; NQN; NOT	09/19/2013
K130021	Philips Herceptest Digital Score	OEO	09/19/2013
K130515	VIRTUOSO System for IHC ER (SPI)	OEO; NQN; NOT	11/22/2013
K131140	Omnyx IDP for HER2 Manual Application	OEO	04/01/2014
K140465	VIRTUOSO System for IHC ER (SP1) with Benchmark Ultra Stainer	OEO; NQN; NOT	03/20/2014
K140957	Genasis HIPATH IHC Family	NQN; NOT	01/15/2015
K141109	Aperio EPATHOLOGY EIHC IVD System	NQN; NOT	07/29/2014
K142965	Virtuoso System for IHC PR (1E2) using iScan HT	OEO	70/16/2015
K172174	Philips IntelliSite Pathology Solution	PSY	10/04/2017
K172922	Barco N.V. MMPC-4127F1 (PP27QHD)	PZZ	12/21/2017
K190332	Aperio AT2 DX System	PSY	05/20/2019
K192259	Philips IntelliSite Pathology Solution	PSY	09/20/2019
K193054	Sectra Digital Pathology Module	QKQ	03/31/2020
K201005	FullFocus	PSY; QKQ	07/15/2020

* *The information listed in this table was collected through searches of FDA's 510 (k) database*.

** *The list is limited to the 6 product codes listed in Table 4*.

In January 2021, a Federal Register (FR) Notice was published suggesting that certain Class I and Class II devices should receive permanent exemption from certain pre-market notification requirements, which included 4 product codes associated with digital pathology products: OEO, PSY, PZZ, QKQ (25). While this FR Notice was ultimately withdrawn in April 2021, citing insufficient information to broadly grant such exemptions (26), this presented a unique opportunity to continue discussions between regulators, industry, and users as multiple public comments to the docket supported a reexamination of the regulatory requirements for digital pathology products. Specifically, the interoperability of the components of these devices, the technical performance assessments, and evidence and studies necessary to bring a product to market now warrant a re-evaluation with the additional experience and new information available on the use of these products. For example, the COVID-19 PHE has presented a unique opportunity to observe the interoperability of certain digital pathology systems in a real-world setting as a result of FDA's guidance enabling the remote use of digital pathology systems that have not undergone 510 (k) pathway clearance (either as a new device or modification to an existing device) for this intended use (27). Industry-leading organizations, such as the DPA, API, and the PIcc continue to engage regulatory bodies in communication on the right-sized requirements needed to introduce digital pathology products to the market to ensure regulation keeps pace with innovation. However, it should also be noted that adoption, not just access, requires additional effort by the field to increase utilization of these practice-enhancing technologies.

This review has outlined several key aspects related to RACA's involvement in TPLC management for digital pathology and AI/ML tools, with the primary aim to establish a common vocabulary to improve communication between the healthcare industry and pathology practice. For industry, the goal was to advocate for increased awareness that many practicing pathologists may be overburdened with the nuanced regulatory terminology. For pathologists, the goal was to help increase understanding of RACA and detangle some of the complexity surrounding it. RACA ultimately bridges the gap between the manufacturers and end users of medical devices, playing a critical role in the TPLC by synthesizing the various components, value propositions, and commercialization of regulated digital pathology solutions in a safe, efficient, and value-based manner.

## Author Contributions

All authors contributed to manuscript generation, revision, read, and approved the submitted version.

## Conflict of Interest

The authors declare that the research was conducted in the absence of any commercial or financial relationships that could be construed as a potential conflict of interest.

## Publisher's Note

All claims expressed in this article are solely those of the authors and do not necessarily represent those of their affiliated organizations, or those of the publisher, the editors and the reviewers. Any product that may be evaluated in this article, or claim that may be made by its manufacturer, is not guaranteed or endorsed by the publisher.
